# A Reply to Dr. C. H. Land’s Critical Review

**Published:** 1885-10

**Authors:** W. B. Ames


### A REPLY TO DR. C. H. LAND'S CRITICAL REVIEW.

BY W. B. AMES, D. D. S.

How Dr. Land, or any fair-minded man could read my paper, published in the July number, and construe it in such a way as to make me an advocate of air chambers, I fail to understand. I very plainly stated, in a paragraph which I will quote, that I did *not* employ them, and I will now say that I would especially avoid the use of an air chamber form, such as I believe my critic has upon the market, that would give more relief over the lateral "interior surface of the mouth" than over the firm surface which we generally find at the central region of the palate. In my paper in the July number, I say:

"Cases have been cited where mouths have been satisfactorily fitted with plain plates, after various failures with plates depending on the suction of a central chamber had been met with, but the citer never describes the relations of his plate to the tissues beneath and about it, which would indicate that a stroke of luck was to be credited with the results. In these cases I hold that something more than the adhesion of contact or capillary attraction must have been secured, either rationally or otherwise, and that this something is a powerful pressure of the atmosphere. This pressure I am able to obtain with plates devoid of any central cavity or air chamber," etc.

That upper artificial dentures can be retained with more satisfaction by utilizing the pressure of the atmosphere after a proper distribution of the bearing (not relief) than by any other method, I have not the shadow of a doubt.

I think that most unprejudiced readers will recognize the conditions which I endeavor to bring about with a denture constructed according to the specifications of my paper. The method of expelling the air from beneath such a denture is not by the action of the muscles about the base of the tongue, as in sucking the air from a lamp chimney, but by merely pressing the denture to its place. Thus, ordinarily, the only pressure manifested is such as overcomes the weight of the denture, plus the weight or pressure of the displaced lax tissues. This pressure, except in the case of a tobacco or gum-chewer, or of a mouth-breather, would be replaced by the pressure of the occluding jaw during a large proportion of the day, so that there is not any injurious traction upon the tissues, except in mastication, or during efforts to displace the denture. It is then, and then only, that the "powerful pressure of the atmosphere" is manifested. The extent of this pressure depends altogether upon the extent and rigidity of the firm tissues covering the jaw.

The simple fact that air is elastic, is for me sufficient reason for its exclusion from beneath the denture to as great an extent as is possible, which I endeavor to accomplish by causing the latter to bear as equally as possible upon all parts of the mouth which it covers.

My reviewer's ideas of air spaces for relief, and an intermediate fluid for retention, are slightly amusing. In the first experiment of the "Review" there is no analogy to the principles which I advanced. The second experiment approaches a representation of the action of such a denture as I construct, but the reviewer apparently does not catch the true light revealed by it. I will suggest that he cement one of his leather disks to a piece of wood, or hard rubber, to the center of which the string is attached, and observe how much adhesion there is between the leather and a moist surface.

I will describe an experiment which is easily conducted, and which will illustrate the action of such a denture as I advocate.

Take an ordinary glass or brass tube of any size, one end of which has been cut at right angles to fit evenly upon a flat surface. Fill this tube with melted beeswax; allow it to harden, and cut from the perfect end a layer of the wax equal in thickness to a piece of heavy rubber dam. Then wet both sides of a piece of the dam, lay it upon a flat surface and press the tube firmly upon it, when it will be found to be held down by a far greater force than that which holds a leather disk of the same size when the string is attached directly to its center. In this experiment the air is expelled from the cavity in the end of the tube by the rubber which filled it, and it is prevented from returning by the close joint formed between the compressed rubber and the edge of the tube, just as the displaced lax tissues form a close joint in the mouth. I think that I have satisfied a few of the profession, before whom I have given clinics, that a long chimney, from which only so much air has been exhausted as will permit it to support one pound to the square inch, does not illustrate all the factors in the problem of atmospheric pressure in the mouth.

